# Prevalence of COVID-19 vaccine hesitancy in Brazil: a systematic review and meta-analysis

**DOI:** 10.3389/fpubh.2025.1622247

**Published:** 2025-08-04

**Authors:** Daniele Melo Sardinha, Marcos Jessé Abrahão Silva, Sebastião Kauã de Sousa Bispo, Alex Patrick Oliveira da Silva, Karla Valéria Batista Lima, Ilma Pastana Ferreira, Luana Nepomuceno Gondim Costa Lima

**Affiliations:** ^1^Postgraduate Program in Epidemiology and Health Surveillance (PPGEVS), Evandro Chagas Institute (IEC), Ananindeua, Brazil; ^2^Postgraduate Program in Parasite Biology in the Amazon (PPGBPA), State University of Pará and Evandro Chagas Institute (UEPA/IEC), Belém, Brazil; ^3^University of the Amazon (UNAMA), Ananindeua, Brazil; ^4^Institutional Scientific Initiation Scholarship Program (PIBIC), Evandro Chagas Institute (IEC), Ananindeua, Brazil; ^5^Postgraduate Program in Nursing, Pará State University (PPGENF/UEPA), Belém, Brazil

**Keywords:** vaccine hesitation, prevalence, public health, COVID-19, Brazil

## Abstract

**Introduction:**

Vaccines have long been one of the most effective strategies for public health during epidemics and pandemics caused by infectious diseases. Vaccine hesitancy is a public health problem that hinders the control of disease transmission. It refers to the refusal or reluctance to receive vaccines for various reasons, including sex, personal beliefs, cultural factors, and a lack of confidence in vaccine safety and efficacy. The COVID-19 pandemic was controlled using vaccines, but maintaining ideal vaccination coverage is essential to continue reducing hospitalizations and deaths from COVID-19. In Brazil, vaccination coverage did not reach the targeted goal, raising concerns about the ongoing impact of COVID-19.

**Objective:**

The study aimed to investigate the prevalence of COVID-19 vaccine hesitancy in Brazil.

**Methodology:**

A systematic review with meta-analysis was carried out using the PubMed, Medline, LILACS, and ScienceDirect databases.

**Results:**

In the search, 238 articles were reviewed, of which 8 were found to be eligible for analysis. The prevalence of vaccine hesitancy was 13.3%, or 0.133 (95% CI = 0.082–0.208), in Brazil. In the subgroup analysis by pandemic wave, the first pandemic wave showed a prevalence of 13.6% or 0.136 (95% CI = 0.081–0.220), while the second pandemic wave had a prevalence of 11.4% or 0.114 (95% CI = 0.029–0.358). Due to heterogeneity, meta-regression showed a significant association between vaccine hesitancy and parents of children and adolescents (*Q* = 95.55; df = 2; *p* = <0.0001), indicating that vaccine hesitancy is significantly higher in this population compared to the general population and older adults. Fake news, lack of knowledge, personal beliefs, income, being a woman, being a young male individual without comorbidities, being married with older children, being older, and being asymptomatic were the main factors influencing vaccine hesitancy.

**Conclusion:**

In the first meta-analysis conducted in Brazil, vaccine hesitancy was more prevalent among parents of children and adolescents, and vaccine hesitancy decreased in the second pandemic wave. Public policies must be developed to address the factors that interfere with acceptance of the COVID-19 vaccine.

## Introduction

Discussions about social health, including worker health and basic sanitary conditions in cities, started during the Industrial Revolution in England. At that time, the world was experiencing multiple epidemics in countries other than Brazil, resulting in thousands of hospitalizations and fatalities due to infectious diseases (1). Hygiene and basic sanitation were the first preventive measures, followed by the introduction of antibiotics and vaccines. Then, the second epidemiological transition occurred, during which infectious diseases were the main cause of illness. Over the decades, cases began to decline, leading to the eradication and elimination of many infectious diseases in several countries and giving rise to chronic diseases as the main cause of illness by the 21st century (2).

To ensure a reduction in hospitalizations and deaths from infectious diseases, vaccines emerged as the primary solution around the world. These immunobiological agents are developed to stimulate the immune response before exposure to pathogens. This development marked a historic milestone, as epidemics were controlled, leading to increased life expectancy. There is no debate about the benefits of vaccines, as we have already seen throughout history that vaccines are the best strategy (3, 4).

To date, challenges to vaccine adherence still exist, with one significant issue being vaccine hesitancy—the non-acceptance of the vaccine due to several factors, including a lack of trust, beliefs, and fake news about possible reactions or complications, such as autism and other diseases (5). Vaccine hesitancy is a public health problem worldwide, as it affects vaccination coverage and collective immunity, thereby jeopardizing the protection of individuals who have contraindications for vaccination. Strategies must be implemented to increase vaccine acceptance, with the aim of preventing epidemics and deaths in the community (6). Dialogue with the local community or through religious leaders, along with the support of health professionals, can help reduce vaccine hesitancy (7).

A pandemic caused by the coronavirus SARS-CoV-2 occurred in 2020 around the world, resulting in significant impacts on health, education, and the economy. The virus caused flu-like symptoms or severe acute respiratory syndrome, which led to millions of hospitalizations and deaths. In response, vaccines were developed in 2020 and rolled out in some countries around the world. In Brazil, the CoronaVac vaccine was administered at the beginning of 2021 to older individuals and healthcare professionals. Throughout 2021 and into 2022, the entire adult population was vaccinated with two doses, while vaccination for children in Brazil only began in 2023. The approved vaccines in Brazil included CoronaVac, Pfizer, Janssen, and AstraZeneca. In 2023, hospitalizations and deaths had already reduced, and restriction measures were being lifted (8–10).

Vaccination coverage with booster doses is still not satisfactory among adults in Brazil. COVID-19 vaccination was included in the vaccination schedule for children; however, coverage remains lower in Brazil among children and adolescents. Low vaccination coverage creates a vulnerability to a potential new COVID-19 epidemic in Brazil and around the world. The Unified Health System (SUS) has successfully distributed the vaccine throughout the country, but vaccine hesitancy remains one of the main factors that prevent ideal vaccination coverage. This hesitancy is due to false reports and denialism propagated by the Brazilian government in 2020 (11, 12).

In Brazil, no meta-analysis has been carried out on COVID-19 vaccine hesitancy. However, epidemiological and analytical studies allow the development of public policies for health promotion. This led to the following research question: “What is the prevalence of COVID-19 vaccine hesitancy in Brazil?”

## Methodology

### Study design

This systematic review and meta-analysis were carried out to investigate the perspectives on and measure the rate of vaccine hesitancy in Brazil. It was carried out following the Preferred Reporting Items for Systematic Reviews and Meta-Analyses (PRISMA) 2020 guidelines to ensure greater transparency and impartiality in the methods applied to this study (13). As this study was classified as a bibliographic review, it did not require ethical approval or committee approval.

### Literature search

To conduct this meta-analysis, a guiding question was developed using the POT strategy (14) (a variation of the PICO anagram)—(P) Problem: COVID-19 vaccination rates in Brazil; (O) Outcome: high levels of vaccine hesitancy for COVID-19; and (T) Type of Study: original epidemiological studies. The objective of this approach was to quantify vaccine hesitancy and understand its determinants, thereby facilitating the development of specific interventions to reduce vaccine resistance in Brazil.

Thus, the following research question was formulated: “What is the prevalence of COVID-19 vaccine hesitancy in Brazil?” Based on this, a search was carried out in international databases for full-text, open-access articles published in Portuguese, English, or Spanish. The search included the descriptors DeCS and MeSH, along with the Boolean operator AND: “Hesitation Vaccination”; “COVID-19”; “Brazil.” The databases selected for this analysis were PubMed, Medline, LILACS, and ScienceDirect, and the time frame considered was from the beginning of the COVID-19 pandemic (March 2020) until the end of the third pandemic wave in Brazil (December 2022) (15, 16).

### Data eligibility

The inclusion criteria for studies were based on original articles with case–control, cross-sectional, cohort, and descriptive epidemiological designs, and research with cross-sectional interviews. The exclusion criteria included incomplete articles, summaries, short communications, letters to the editor, case studies, case series, studies with unavailable data for extraction, and studies published in languages other than those selected for this research. Gray literature was excluded to ensure the highest level of evidence in epidemiology, considering the peer review and methodological quality of the articles published in scientific journals.

### Data extraction and assessment of the methodological quality of the studies

After selection through independent and paired reading by two researchers (DMS and MJAS) and discussion of ideas with a third researcher (SKSB), the articles were organized in a Microsoft Office Excel 365 spreadsheet. Data extracted from the original documents included the article title, methodology, the database used, the pandemic wave referenced (based on epidemiological criteria for pandemic waves in Brazil), vaccine hesitancy rates, and the factors associated with the issue addressed in each study.

Then, a methodological assessment of the quality of the studies to be included was carried out through paired and independent selective assessments by two researchers (DMS and MJAS), with discussions involving a third researcher (SKSB) when needed. The assessments were based on the standardized checklists provided by the Joanna Briggs Institute (JBI), which are commonly used in systematic reviews to ensure data reliability. The JBI critical appraisal tools are instruments that are used to assess the trustworthiness, relevance, and results of published articles (17). The checklists used in your scoring form electives were as follows: Checklist for Analytical Cross-Sectional Studies, Checklist for Case–Control Studies, Checklist for Cohort Studies, Checklist for Prevalence Studies, and Checklist for Qualitative Research (18). Only studies that met more than 60% of the checklist requirements were selected, in accordance with the methodology outlined in previous literature (19).

### Statistical analysis

Statistical analysis was conducted using Comprehensive Meta-Analysis v3.3 software (Biostat, Englewood, NJ, USA), with a proportion meta-analysis performed to estimate the prevalence. A random effects model was used due to the contextual discrepancies in each pandemic period and the different factors associated with possible outcomes of vaccine hesitancy, even within the same population assessed (20). A subgroup analysis was carried out according to the pandemic wave in each study. In addition, heterogeneity was assessed, a funnel plot was used to evaluate the risk of publication bias, and meta-regression was performed based on the type of population analyzed in each article (21).

## Results

### Study selection

**Figure 1:**
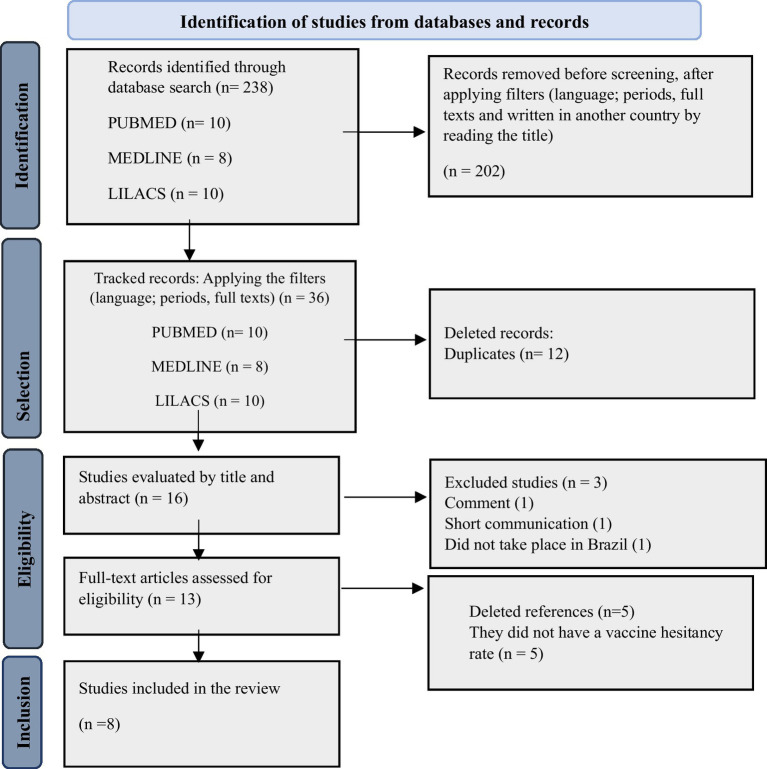
PRISMA flow diagram (2020) illustrating the selection of the studies included in this review. Source: authors’ own elaboration (2024).

A search of the selected databases yielded 238 articles: 10 from PubMed, 8 from Medline, 10 from LILACS, and 10 from ScienceDirect. After applying the filters (language, study period, availability of full texts, and studies conducted in Brazil), 36 studies remained. These studies were screened for titles related to vaccine hesitancy in Brazil. Duplicate studies were excluded (*n* = 12). In this context, we evaluated 16 complete articles. Furthermore, one article was excluded because it was a commentary, another was excluded for being a short communication (*n* = 1), and a third article was excluded since it evaluated several countries but did not include Brazil (*n* = 1). Then, a total of 13 studies were evaluated for data extraction and inclusion in our sample. After careful reading and analysis, five studies were excluded for not presenting the vaccine hesitancy rate, and eight studies (22–29) were eligible to be included in the sample for this systematic review (Figure 1).

### Characterization of the included studies

A total of eight studies were included in the analysis. The studies were published in 2021 (2), 2022 (3), and 2023 (3). Seven studies were in English, and one was in Portuguese. Two (2) (23, 29) studies were related to the first pandemic wave, while the remaining six (6) (22, 24–28) focused on the second pandemic wave. The majority of the studies were sourced from the PubMed and Medline databases (*n* = 4; 50%). Regarding the populations studied, six studies evaluated the general population (22, 23, 25, 27–29), one study focused on older people (26), and one study examined parents of children and adolescents (24). The factors associated with vaccine hesitancy included being a woman, not being afraid of developing COVID-19, having been affected by COVID-19, political issues, belonging to a wealthier class, concerns about adverse reactions to, distrust of the vaccine, denial about the pandemic, receiving vaccine information from friends, family, and social networks, not having received the influenza vaccine, young male individuals without comorbidities, cases with older children, older people, evangelicals, and those who were asymptomatic (Table 1). Only three studies deviated from the maximum score on the JBI quality checklist. Of the six cross-sectional studies, two had a score of 87.5%, and of the two descriptive studies, one had a descriptive score of 90%.

**Table 1:** Characteristics of the studies included in this meta-analysis.

Title	Methodology/sampling	Pandemic wave	Database	Vaccine Hesitation	Associated Factors	JBI score (*)
Low COVID-19 vaccine hesitancy in Brazil	Cross-sectional survey / 173,178 participants	1st pandemic wave	ScienceDirect	10.5%	Having little or no fear of developing COVID-19 and believing that the vaccine was unnecessary for individuals who had already been infected with COVID-19.	7/8
Prevalence, predictors, and reasons for COVID-19 vaccine hesitancy: results of a global online survey	Cross-sectional survey/15,536 participants	2nd pandemic wave	ScienceDirect	15%	Political view is a predictor of vaccine hesitancy, with greater hesitancy observed as one moves further to the political right. Furthermore, a positive socioeconomic gradient in hesitancy was observed, with greater hesitancy observed in individuals from wealthier socioeconomic classes.	8/8
Online survey on the reasons for vaccine hesitancy against COVID-19 in children and adolescents in Brazil	Descriptive 1,896 participants	2nd pandemic wave	Medline and PubMed	87%	Fears about vaccination due to the experimental nature of the vaccine, concerns about adverse reactions, and potential long-term effects.	9/10
Social Representations of Hesitant Brazilians about Vaccination against COVID-19	Descriptive 173,178 participants	2nd wave of the pandemic	Medline and PubMed	6.30%	Distrust of the vaccine, underestimation of the severity of the pandemic, misinformation, distrust of political involvement, and fear of adverse reactions to COVID-19 vaccines.	10/10
COVID-19 vaccine hesitancy in a national sample of older Brazilians: the ELSI-COVID Initiative, March 2021	Cross-sectional 4,364	2nd wave of the pandemic	Medline and PubMed	2.50%	Individuals who found out about COVID-19 through friends/family/social media were more likely to be undecided about vaccination (odds ratio = 3.15; 95%CI 1.28; 7.77).	8/8
COVID-19 vaccine hesitancy and associated factors according to sex: a population-based survey in Salvador, Brazil	Cross-sectional 2,521	2nd wave of the pandemic	Medline and PubMed	18.6%	Women (79.7%; 95% CI 77.7–81.6%) were an associated factor, as were younger men without comorbidities. In general, individuals who did not receive the influenza vaccine were also hesitant about the COVID-19 vaccine.	7/8
Intention to get vaccinated against COVID-19 and vaccine hesitancy in Southern Brazil: prevalence and associated factors	Cross-sectional 953	1st wave of the pandemic	LILACS	3.9%	Vaccine hesitancy was associated with being married, having children, and being older.	8/8
Prevalence and factors associated with COVID-19 vaccine hesitancy in Maranhão, Brazil	Cross-sectional 4,630	1st wave of the pandemic	LILACS	17.5%	Vaccine hesitancy was statistically higher among women (19.8%; PR = 1.44; 95%CI 1.20–1.75), older adults (22.8%; PR = 1.79; 95%CI 1.30–2.46), evangelicals (24.1%; PR = 1.49; 95%CI 1.24–1.79), and those without reported symptoms (18.6%; PR = 1.24; 95%CI 1.02–1.51).	8/8

Source: authors’ own elaboration (2024).

### Meta-analysis results and publication bias

**Figure 2:**
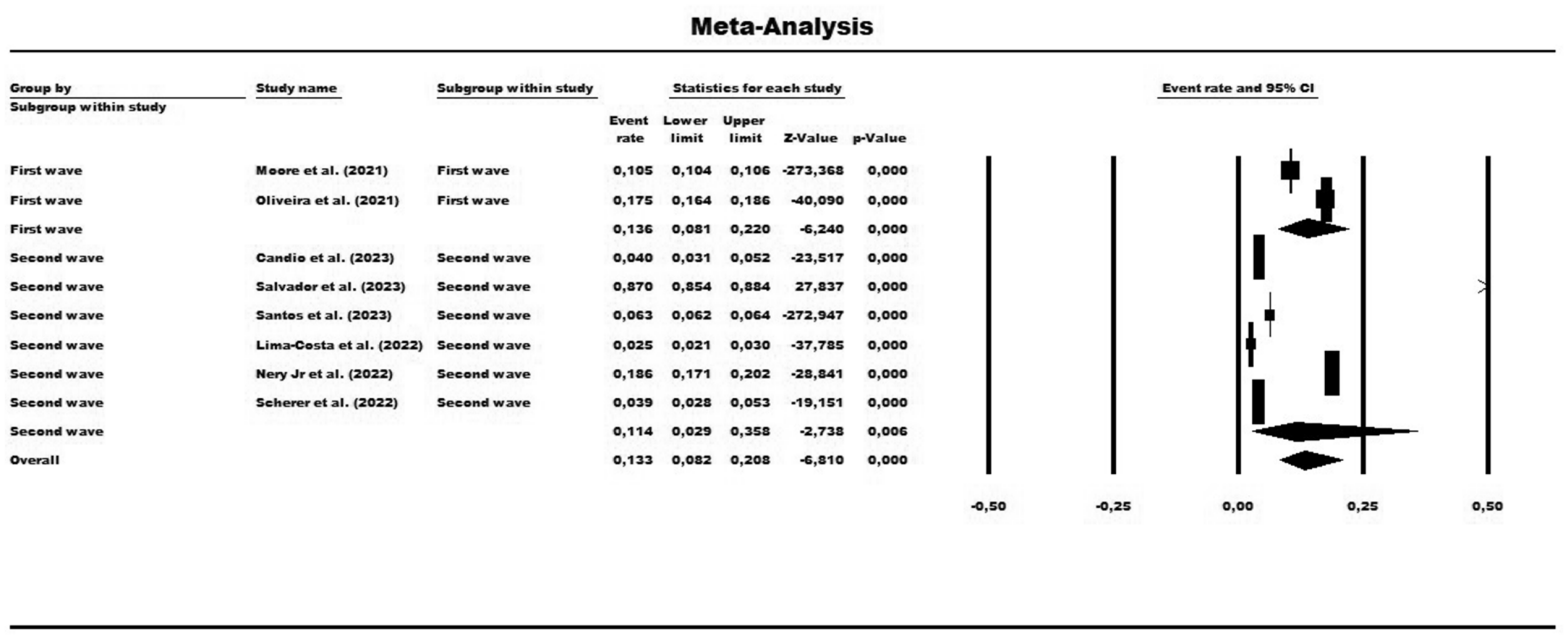
Forest plot showing the prevalence of COVID-19 vaccine hesitancy for the total sample, grouped by subgroups and pandemic waves. The size of each square corresponds to the weight of the related research in the meta-analysis, representing the odds ratio (OR) of each study on the map. The 95% confidence intervals (CIs) for the OR of each study are shown as horizontal lines. Numbers in bold indicate the overall OR and 95% CI, along with the total number of cases and controls.

**Figure 3:**
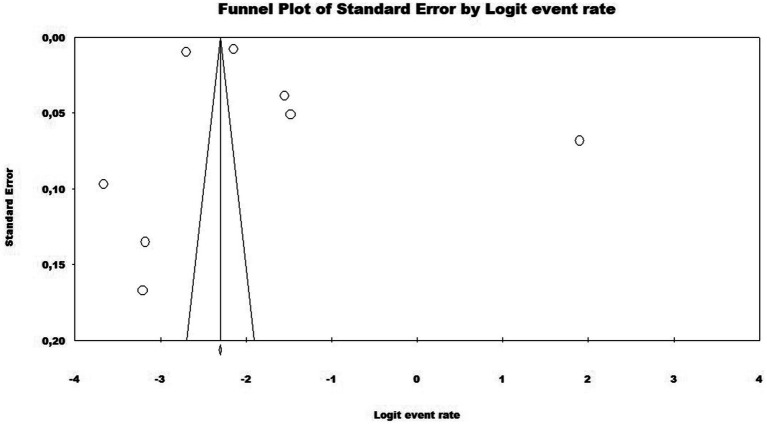
Funnel plot assessing publication bias in the meta-analysis of the prevalence of vaccine hesitancy regarding the SARS-CoV-2 vaccine in Brazil.

In the analysis of these eight studies using a random effects model, the prevalence rate of COVID-19 vaccine hesitancy in Brazil was 13.3% or 0.133 (95% CI = 0.082–0.208). The disparities between the analyses showed statistically significant heterogeneity (*Q*-value = 6721.818; Df = 7; I ^2^ = 99.89%), with heterogeneity highlighted by the high value of I^2^, even within subgroups of pandemic waves (first wave = 99.55%; second wave = 99.90%). In the subgroup analysis by pandemic wave, the first pandemic wave showed a prevalence of 13.6% or 0.136 (95% CI = 0.081–0.220), while the second pandemic wave had a prevalence of 11.4% or 0.114 (95% CI = 0.029–0.358) (Figure 2). The distribution was symmetrical in the funnel plot, indicating low publication bias in the meta-analysis (Figure 3).

### Meta-regression of the included studies through the population type covariate

**Figure 4:**
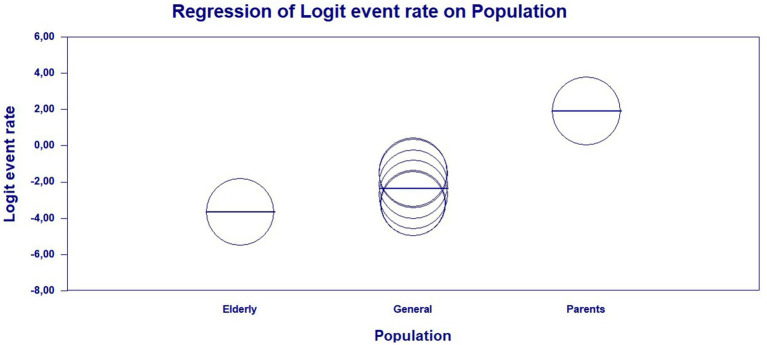
Scatterplot of the meta-regression of the event rate by study population type—general population, older adults, and parents of children and adolescents. Bubble size is inversely associated with study variance. The solid line reflects linear regression.

Due to the high level of heterogeneity, it was necessary to search for the source of the factor that likely had the greatest weight in the concentration of divergent data. Therefore, a meta-regression was performed. The results of the meta-regression, using the type of study population as a moderator, showed a significant association between vaccine hesitancy and parents of children and adolescents (*Q* = 95.55; df = 2; *p* = <0.0001), indicating that vaccine hesitancy in this population is much higher compared to the general population and older adults (Figure 4 and Table 2).

**Table 2:** Main results of Model 1, random effects (MM), *Z*-distribution, and logit event rate.

Covariate	Coefficient	Standard	95%	95%	*Z*-value	2- sided	Set
		Error	Lower	Upper		*p*-value	
Intercept	−3.6636	0.4469	−4.5394	−2.7877	−8.2	0	
Population: general	1.3157	0.4824	0.3702	2.2612	2.73	0.0064	*Q* = 95.55, df = 2, *p* < 0.0001
Population: parents	5.5645	0.6282	4.3333	6.7958	8.86	0	*Q* = 95.55, df = 2, *p* < 0.0001

Statistics for Model 1. Test of the model: Simultaneous test of all coefficients (excluding intercept) being zero: *Q* = 95.55, df = 2, *p* = 0.0000. Goodness of fit: Test that evaluates whether the unexplained variance is zero: Tau^2^ = 0.1903, Tau = 0.4362, I^2^ = 99.82%, *Q* = 2716.97, df = 5, *p* = 0.0000. Comparison of Model 1 with the null model. Total between-study variance (intercept only): Tau^2^ = 0.4560, Tau = 0.6753, I^2^ = 99.90%, *Q* = 6721.82, df = 7, *p* = 0.0000. Proportion of the total between-study variance explained by Model 1: *R*^2^ analog = 0.58.

## Discussion

This is the first meta-analysis to investigate the prevalence of COVID-19 vaccine hesitancy in Brazil. The limited number of available studies highlights the need for further research in this field, as vaccine hesitancy factors must be explored to guide public policies aimed at improving access and adherence to COVID-19 vaccination.

The overall prevalence of COVID-19 vaccine hesitancy in this meta-analysis was 13.3%. We analyzed vaccine hesitancy by pandemic wave and observed that in the first year of the pandemic, vaccine hesitancy was higher at 13.6%, while in the second year of the pandemic, it was 11.4%. However, meta-regression showed that the greatest difference in vaccine hesitancy was among parents of children and adolescents, compared to the general population and older adults.

Several factors affect adherence to the COVID-19 vaccine, including ethnicity, professional status, religious beliefs, political views, sex, age, education, and income (30). One study reported COVID-19 vaccine acceptance rates in various countries, with the highest rates observed in Ecuador (97.0%), Malaysia (94.3%), Indonesia (93.3%), and China (91.3%). The lowest rates were also found in Kuwait (23.6%), Jordan (28.4%), Italy (53.7%), Russia (54.9%), Poland (56.3%), the United States (56.9%), and France (58.9%) (31).

A systematic review conducted in the United States showed that the overall vaccine acceptance rate ranged from 12 to 91.4%. Factors associated with vaccine hesitancy were sex, ethnicity, age, education level, and income status. In addition, vaccine acceptance increased by 10.8% in 2020 and 7.4% in 2021. Vaccine hesitancy in the U. S. population was the highest among Black/African Americans and pregnant or breastfeeding women, while it was the lowest among men (32). Another study in the United States found that lack of confidence in the vaccine accounted for 38 and 21% of the variation in vaccine hesitancy. Sociodemographic and psychological determinants were responsible for 13 and 11% of the variation in vaccine hesitancy (33).

Another study conducted in Portugal showed that 56% of participants would wait before taking the COVID-19 vaccine, and 9% would refuse the COVID-19 vaccine. The associated factors included being younger, loss of income, not taking the flu vaccine, low confidence in the COVID-19 vaccine and the health services’ response, and doubts about the effectiveness of the vaccine (34).

Research has shown that factors such as anxiety and fear concerning one’s own health significantly influence vaccine acceptance, while fear of social and economic consequences is associated with vaccine hesitancy (35). A review highlighted several factors associated with COVID-19 vaccine hesitancy, such as concerns about vaccine effectiveness, side effects, distrust, religious beliefs, and trust in information sources (36). A study conducted in Türkiye during the first year of the pandemic found that 45.3% of participants were hesitant to receive the COVID-19 vaccine. The factors that influenced vaccine acceptance were risk perception (OR: 1.26% IC95 1.03–1.55) and age (OR: 0.94% IC95: 0.91–0.98) (37).

Potential sources of heterogeneity in Brazil may stem from various contexts. Cultural, regional, psychological, economic, religious, political, cognitive, and sex-related considerations contribute to the complex behavior surrounding vaccination decisions. COVID-19 vaccine hesitancy in Brazil is strongly influenced by the sociopolitical context of the country, characterized by a combination of misinformation, political polarization, and inequalities within the healthcare system. The context of social and regional inequalities also shapes the hesitancy, with specific religious groups, such as evangelicals, showing greater resistance, possibly due to cultural and religious convictions that affect vaccine acceptance (23, 29).

Another study conducted in Arkansas during the first year of the pandemic showed that vaccine hesitancy was higher among Black/African Americans (50.00%), individuals with a family income below US$25,000 (30.68%), those with some higher education (32.17%), those with little or no fear of COVID-19 infection (62.50%), and those with low confidence in vaccines in general (55.84%) (38). A study conducted in European countries during the second year of the pandemic highlighted that COVID-19 vaccine hesitancy was lowest in Spain, at 6.22% for women and 6.82% for men, compared to Bulgaria, where the rates of hesitancy were higher, at 64.19% for women and 59.20% for men. Men showed higher vaccine acceptance than women in the countries analyzed (39). Heterogeneity was associated with sex and groups, such as income and ethnicity.

In our meta-analysis, parents of children and adolescents showed the greatest heterogeneity among the groups, with vaccine hesitancy rates higher than those observed in other populations. This is concerning, as children and adolescents depend on their parents to get vaccinated. In Switzerland, it was no different, where an online survey study on the acceptance of the COVID-19 vaccine by country in relation to children found that the intention to vaccinate children was 58.7%. This indicates that the proportion of those unwilling to vaccinate remained high, even in the second year of the pandemic (40).

Another study in Jordan highlighted that 21.5% of parents were unsure about vaccinating their children, while 48.4% intended not to vaccinate them (41). A survey of mothers in the United Kingdom, conducted during the second year of the pandemic, showed vaccine hesitancy regarding their children in two groups: 38.4% in the first group and 25.6% in the second (42). In Qatar, a survey conducted during the first year of the pandemic showed that 29.4% of mothers were hesitant to vaccinate their children against COVID-19, while 27.5% expressed personal vaccine hesitancy. The highest hesitancy rate was associated with ethnicity, with 51.3% of Qatari mothers reporting hesitancy (43). A study in Shenzhen, China, reported a hesitancy rate of 43.37% (44). Similarly, in Saudi Arabia, 49.4% of parents did not wish to immunize their children with the COVID-19 vaccine, and 12.8% were unsure (45).

A survey of COVID-19 vaccination coverage in Norwegian children and adolescents conducted in December 2022 included 423,548 children aged 5 to 11 years, 269,830 children aged 12 to 15 years, and 120,854 children aged 16 to 17 years. Vaccine adherence in these three groups was 2.6, 73.3, and 87.3%, respectively (46). The country’s high vaccine hesitancy is responsible for low vaccination coverage among children and adolescents.

One of the reasons for the country’s general vaccine hesitancy was the association of autism spectrum disorder (ASD) with vaccination. However, this is a myth—false news shared on social media—that has contributed to low vaccination coverage worldwide. It has already been proven through extensive research that there is no association between ASD and vaccines in children and adolescents (47).

In Brazil, the “Observa Infância” project analyzed data on COVID-19 vaccination from February 2024. Vaccination coverage among children aged 3 to 4 years was 23% for two doses and only 7% for the complete vaccination schedule with three doses. In the age group of 5 to 11 years, coverage increased to 55.9% for two doses and 12.8% for completing the three-dose regimen (48). The low vaccination coverage in the fourth year of the pandemic in Brazil among children and adolescents is a public health problem. Strategies should focus on reducing vaccine hesitancy among parents. A lack of knowledge about vaccines is one of the main reasons for hesitancy, with indecision and lifestyle factors also influencing this decision (49).

The limitations of this review include high heterogeneity, possible publication bias, limited data on certain subpopulations, and potential misclassification of hesitancy levels. The main limitation of this meta-analysis is the small number of studies carried out in Brazil and the heterogeneity identified through the meta-regression. Therefore, future studies on vaccine hesitancy should be prioritized in Brazil to support public health planning and achieve an acceptable vaccination coverage of at least 95%. In addition, the COVID-19 pandemic occurred during a period of extreme political polarization in Brazil, which was accompanied by widespread circulation of fake news and contradictory information, even from health professionals and authorities. This fragmented and conflicting communication and high scientific denialism contributed to distrust of the vaccine and an increase in vaccine hesitancy (50).

Regarding the relationship between COVID-19 vaccine hesitancy and mortality/morbidity in Brazil, it was revealed that this lethality was linked to inadequate vaccination coverage, as vaccine hesitancy (27.5% nationwide) directly hindered herd immunity and permitted the spread of severe outcomes (51). Regional differences were striking, while scientific denialism and mistrust of health officials intensified transmission, and towns that voted for then-President Bolsonaro in the 2018 election experienced significantly higher mortality rates during the second wave (52, 53). In communities influenced by evangelical missionaries, conspiracy theories—such as untrue claims that vaccines cause people to change into “alligators”—became popular (54, 55). Bolsonaro’s public skepticism—including derision toward vaccines such as Sinovac and Pfizer—amplified distrust among his supporters. Structural equation modeling demonstrated that reluctance was inversely correlated with faith in governmental entities (54, 56). Due to this politicization, vaccination became a partisan issue, and people on the political left showed greater compliance (54, 57, 58).

Therefore, for future public health policies addressing Brazil’s inequalities, studies have shown that there is a need to depoliticize vaccination through cross-partisan campaigns that isolate health guidelines from political narratives, personalize community engagement to combat misinformation in religious and indigenous communities by collaborating with trusted religious leaders, prioritize regional equity by aligning genomic surveillance and vaccine access with socioeconomic vulnerability indicators, and strengthen institutional trust through transparent communication about vaccine safety and by directly countering conspiratorial beliefs. These measures must be recognized among Brazil’s sociopolitical fractures to prevent recurring mortality disparities during health crises (59, 60).

## Conclusion

This is the first meta-analysis on vaccine hesitancy against COVID-19 in Brazil, and it showed that hesitancy prevalence was higher during the first year of the pandemic and decreased during the second year. The significant variation between the studies was strongly influenced by the specific population evaluated, with emphasis on the high level of vaccine hesitancy among parents of children and adolescents.

Public policies need to be developed to address the factors that interfere with the acceptance of the COVID-19 vaccine, especially among parents of children and adolescents in Brazil. Fake news, lack of knowledge, beliefs, income level, female sex, young male individuals without comorbidities, being married with older children, older age, and being asymptomatic are the main factors influencing vaccine hesitancy.

## Data Availability

The original contributions presented in the study are included in the article/supplementary material, further inquiries can be directed to the corresponding author.
